# Transcription factor-based classification of pituitary adenomas / PitNETs: a comparative analysis and clinical implications across WHO 2004, 2017 and 2022 in 921 cases

**DOI:** 10.1186/s40478-025-02050-8

**Published:** 2025-06-28

**Authors:** Isabella Nasi-Kordhishti, Mirko Hladik, Kosmas Kandilaris, Felix Behling, Jürgen Honegger, Jens Schittenhelm

**Affiliations:** 1 Department of Neurosurgery and Neurotechnology, University Hospital Tübingen, Eberhard-Karls-University Tübingen, Hoppe-Seyler-Strasse 3, 72076 Tübingen, Germany; 2 Department of Orthopedic, Trauma and Spine Surgery, Thun Hospital, Thun, 3600 Switzerland; 3 Department of Neuropathology, University Hospital Tübingen, Eberhard-Karls-University Tübingen, 72076 Tübingen, Germany; 4 Faculty of Medicine, Medical Center, Institute for Surgical Pathology, Albert Ludwig University of Freiburg, 79098 Freiburg, Germany; 5 Center for Neuro-Oncology, Comprehensive Cancer Center Tübingen-Stuttgart, University Hospital Tübingen, Eberhard-Karls-University Tübingen, 72076 Tübingen, Germany; 6 Hertie Institute of Clinical Brain Research, University Hospital Tübingen, Eberhard-Karls-University Tübingen, 72076 Tübingen, Germany

**Keywords:** WHO classification, Pituitary transcription factors, PIT-1, T-PIT, SF-1, Pituitary adenoma, PitNET

## Abstract

The WHO classifications of 2017 and 2022 recommend the use of pituitary transcription factors PIT-1, T-PIT and SF-1 as well as GATA3 and ERα for histopathological diagnosis. The aim of this study is to demonstrate their diagnostic impact in a large retrospective cohort. 921 PitNETs/PAs diagnosed in our department between October 2004 and April 2018 were retrospectively reassessed according to the WHO classifications 2017 and 2022. The original classification (WHO 2004) and the clinical data were retrieved from the patient records. Hormone-immunonegative null cell adenomas represented the largest subgroup (397 of 921) in the WHO 2004 classification. Of these, 377 were reclassified as gonadotroph PitNETs/PAs, and 14 were assigned to a non-gonadotroph hormone-producing cell line. Only 6 cases remained null cell tumors. 27 of 35 plurihormonal adenomas were assigned to a specific cell line in the 2017 and 2022 WHO classifications. Of 489 adenomas formerly classified as expressing of 1 or 2 hormones, the histopathological diagnosis was confirmed in 459 cases with the use of TP. Of the remaining 30 cases, 12 cases with positive immunostaining of 2 hormones could be assigned to a single cell line, and 18 cases changed their lineage. The correct correlation with clinical data significantly improved from 75.4% (WHO 2004) to 96.2% (WHO 2017 and 2022). Corticotroph PitNETs showed the highest risk for recurrence (21.9%) and progression (55.8%). The new classification enables more accurate (sub)classification and significantly improves clinicopathological correlation. In individual cases, it is essential to consider the reclassification to predict the clinical prognosis and to schedule the follow-up accordingly.

## Introduction

The classification of pituitary neuroendocrine tumors (PitNET), previously termed pituitary adenomas (PA), has changed significantly in recent years. Initially, they were divided into 3 cell groups based on conventional light microscopy: acidophil (somatotropic axis), basophil (corticotropic axis) and chromophobic (gonadotroph lineage). From the beginning of the 1990s, immuno-histochemistry was increasing used for further delineation [1]. The World Health Organization (WHO) classification of 2004 was based on immunostaining for pituitary hormone expression and ultrastructural features of pituitary adenomas [2, 3]. Subsequently, immunohistochemical examination of specific transcription factors (TF) was introduced, thereby enabling the identification of the original cell line of PAs/PitNETs. The pituitary-specific transcription factor 1 (PIT-1) has been associated with somatotroph, thyrotroph and lactotroph lineages [4–6], the t-box transcription factor (T-PIT) with the corticotroph lineage [7, 8] and the steroidogenic factor-1 (SF-1) with the gonadotroph lineage [9, 10]. The WHO classification of 2017 recommends the use of the three main pituitary TF (PIT-1, T-PIT and SF-1) in the histopathological diagnosis [11–14]. From a neuropathological perspective, this has led to a notable shift in the distribution of PitNETs/PAs to specific types and subtypes, with a marked reduction in the prevalence of null cell adenomas [15, 16]. The WHO classification of 2022 introduced the term “PitNET” as a new terminology for “PA” [17]. It added further refinements among others replacing PIT-1 positive plurihormonal tumor by two clinicopathologically distinct PitNET, namely the immature PIT-1-lineage tumor (formerly known as silent subtype 3 tumor) and the mature PIT-1-lineage tumor (Table 1) [11, 18].

**Table 1:** Overview WHO classification of 2017 and 2022 [11, 18]

WHO 2017			WHO 2022		
Transcription factor	Adenoma type	Subtype	Transcription factor	PitNET type	Subtype
PIT-1			PIT-1		
	*Somatotroph adenoma*	*DGSA*		*Somatotroph tumors*	*DGST*
		*SGSA*			*SGST*
		*MSA*		*Lactotroph tumors*	*DGLT*
		*MSLA*			*SGLT*
	*Lactotroph adenoma*	*DGLA*		*MST*	
		*SGLA*		*MSLT*	
		*ASCA*		*Thyrotroph tumor*	
	*Thyrotroph adenoma*			*Mature PIT-1-lineage tumor*	
		*Immature PIT-1-lineage tumor*			
		*ASCT*			
T-PIT			T-PIT		
	*Corticotroph adenoma*	*DGCA*		*Corticotroph tumors*	*DGCT*
		*SGCA*			*SGCT*
		*Crooke cell*			*Crooke cell*
SF-1			SF-1		
	*Gonadotroph adenoma*			*Gonadotroph tumor*	
None			PitNETs with no distinct cell lineage		
	*Null cell adenoma*			*Plurihormonal tumor*	
				*Null cell tumor*	
Other					
	*Plurihormonal adenoma*	*Plurihormonal PIT-1 positive adenoma*			
		*Adenoma with unusual immunohisto-chemical combination*			

DGSA: dense granulated somatotroph adenoma; SGSA: sparsely granulated somatotroph adenoma; MSA: mammosomatotroph adenoma; MSLA: mixed somatotroph and lactotroph adenoma; DGLA: dense granulated lactotroph adenoma; SGLA: sparsely granulated lactotroph adenoma; ASCA: acidophil stem cell adenoma; DGCA: dense granulated corticotroph adenoma; SGCA: sparsely granulated corticotroph adenoma; DGST: dense granulated somatotroph tumor; SGST: sparsely granulated somatotroph Tumor; MST: mammosomatotroph tumor; MSLT: mixed somatotroph and lactotroph tumor; DGLT: dense granulated lactotroph tumor; SGLT: sparsely granulated lactotroph tumor; ASCT: acidophil stem cell tumor; DGCT: dense granulated corticotroph tumor; SGCT: sparsely granulated corticotroph tumor

The aim of this study is to demonstrate how the innovations of the 2017 and 2022 WHO classifications influenced the classification of PAs/PitNETs in a large retrospective cohort. Both the shift of assignment to PitNETs/PAs subtypes and the clinical implications were analysed.

## Materials and methods

### Patient cohort

We retrospectively analyzed all tissues of PAs/PitNETs that were surgically treated at our pituitary center between October 2004 and April 2018. During this period, the classification of all cases was conducted in accordance with the WHO classification 2004, and all specimens were collected and processed uniformly. A total of 1791 sellar pathologies were identified, of which 452 were excluded due to an entity other than PA/PitNET. Of the remaining 1339 PA/PitNET cases, 1296 were eligible for study inclusion and construction of tissue microassays (TMA) for high-throughput retrospective analysis. After examining archival paraffin blocks, 375 cases did not have sufficient tumor tissue left, or the existing vital tumor sample was not suitable (i.e. extensive bleeding or necrosis). Consequently, tissue samples from 921 tumors were constructed into TMA blocks. The original classification according to the WHO classification 2004 and the clinical data were retrieved from the patient records. None of these 921 tumors was initially stained for PIT-1, T-PIT and SF-1. All cases were retrospectively reassessed by applying the missing pituitary TF stains, and also estrogen receptor (ERα), GATA3 and keratin (CAM5.2) stains.

### Tissue microarray and immunohistochemistry

The optimal area for TMA was identified in each tumor tissue sample by experienced neuropathologists and marked on the corresponding haematoxylin and eosin (HE) stains. Two regions of interest from each sample were biopsied with 1000 μm cylinders and rearranged on a donor recipient block using a conventional tissue microarrayer (Beecher Instruments, Sun Prairie, Wisconsin, USA). In tumors with heterogeneous or plurihormonal hormone expression, the selected regions reflected the dominant histological and immunohistochemical features based on the original 2004 diagnosis. In most cases, hormone expression was evenly distributed across the tumor parenchyma, allowing for consistent selection. However, in one case with a double PA/PitNET, distinct compartments with different hormone profiles were identified. In this instance, two separate regions were sampled from each tumor. From each sealed TMA, twenty 3 μm thin slices were cut and fixed on glass slides with a negative charge. Subsequently, immunohistochemical staining was conducted using a Roche Benchmark XT immunohistochemistry system for PIT-1, T-PIT, SF-1, GATA3, ERα and Cam5.2 keratin with identical settings for routine diagnostic neuropathology. The stained TMA slides were then examined microscopically to ascertain the presence of tumor tissue and the expression of TF, ERα, GATA3 and keratin staining patterns. The stains ware assessed semi-quantitatively by the neuropathologists. The samples were reclassified according to the WHO classification 2017 and then 2022.

### Statistical methods

Statistical analysis was done using JMP^®^ Version 17 (SAS Institute Inc.; Cary, NC). Descriptive data is presented as mean, standard deviation (SD), and percentage. Group differences were evaluated by one-way analysis of variance (ANOVA). For each statistical test, results were considered to be statistically significant if the p-value was < 0.05. Recurrence and progression-free survival was estimated using the Kaplan-Meier method. The length of follow-up (FU) for recurrence and progression-free survival was calculated from the date of surgery to the date of recurrence or the last clinical visit.

### Image Preparation

Sankey diagrams were created using SankeyMATIC (Freeware available software at: https://sankeymatic.com/build/) [19]. Stained slides were scanned using a Zeiss Mirax slide scanner (Zeiss; Göttingen, Germany), and the resulting images were taken as screenshots using the Mirax Viewer software (Zeiss; Göttingen, Germany).

## Results

### Histopathological types/subtypes according to the WHO classification 2004

The 921 cases were initially diagnosed according to the WHO classification 2004 (Table 2), which is essentially based on immunostaining of hormone expression, namely adrenocorticotrophic hormone (ACTH), follicle stimulating hormone (FSH), human growth hormone (GH), luteinising hormone (LH), prolactin (PRL) and thyroid stimulating hormone (TSH). A total of 397 cases (43.1%) were hormone-immunonegative or showed only single cells with hormone expression. These cases were classified as (hormone-immunonegative) null cell adenomas. The remaining 524 cases (56.9%) were immunopositive. Of these, 346 cases (37.6%) showed distinct expression of a single hormone, with GH- and ACTH-producing adenomas being most prevalent. Expression of two hormones was found in 143 cases (15.5%). Among these, the combination of GH and PRL expression was predominantly observed (*n* = 97). The remaining 35 cases (3.8%) were classified as plurihormonal adenomas, characterized by the expression of more than two pituitary hormones (Table 2).

**Table 2:** Distribution according to WHO classification 2004

Hormone Expression	*N*=	%
HGH	109	11.8
HGH + ACTH	5	0.5
HGH + TSH	3	0.3
HGH + PRL	97	10.5
PRL	72	7.8
PRL + ACTH	4	0.4
PRL + FSH	2	0.2
PRL + TSH	3	0.3
TSH	7	0.8
ACTH	99	10.8
ACTH + FSH	2	0.2
ACTH + LH	1	0.1
FSH	38	4.1
LH	21	2.3
LH + FSH	26	2.8
Nell-cell-adenoma	397	43.1
Plurihormonal	35	3.8

ACTH: adrenocorticotrophic hormone; FSH: follicle stimulating hormone; HGH: human growth hormone; LH: luteinising hormone; PRL: prolactin; TSH: thyroid stimulating hormone

### Histopathological types/subtypes according to the WHO classification of 2017 and 2022

**Fig. 1:**
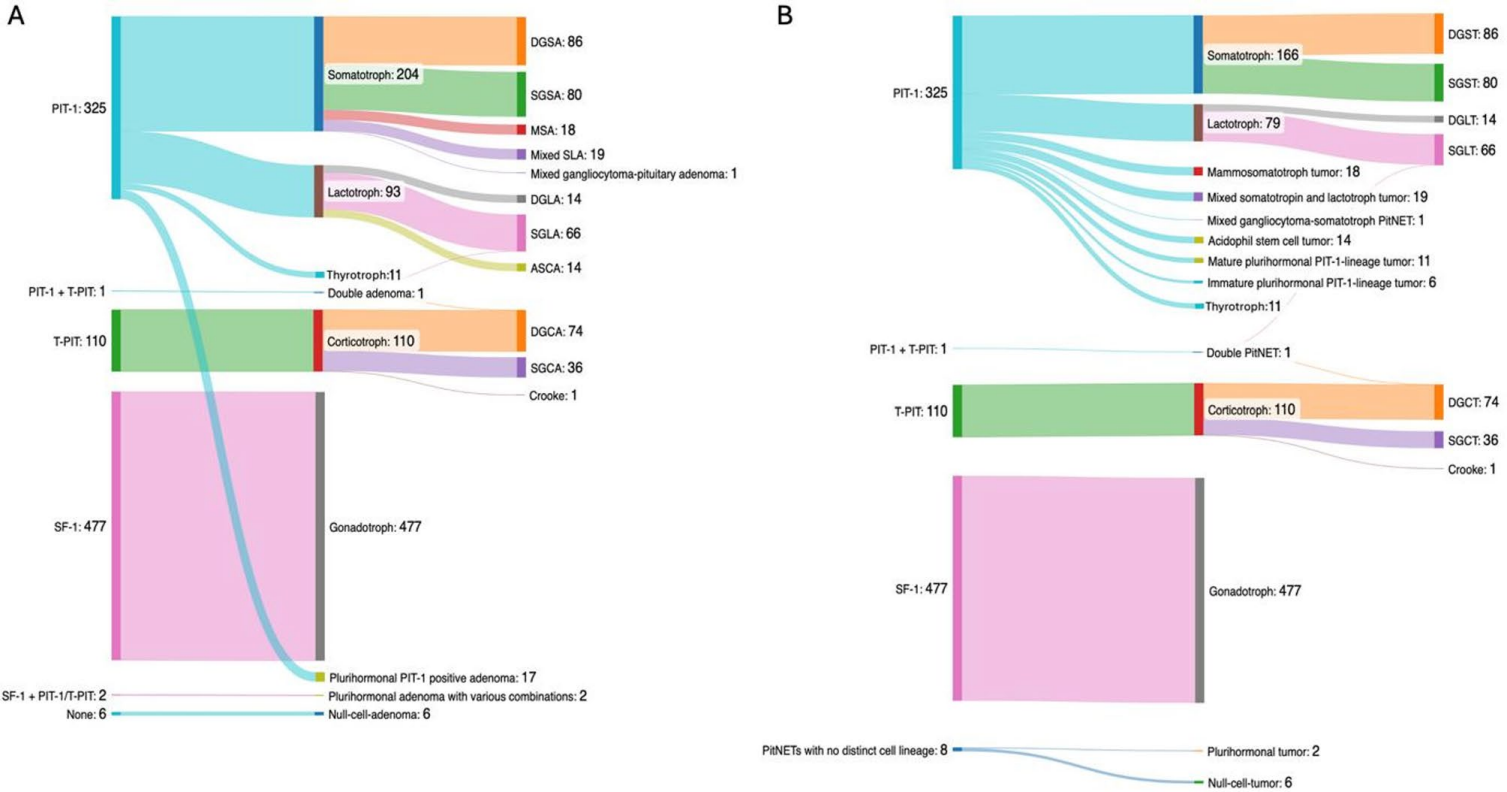
Distribution according to WHO classification 2017 (**A**) and 2022 (**B**)

Following immunostaining with the specific pituitary TF according to the WHO classifications 2017 and 2022, 325 cases (35.3%) were positive for PIT-1, 110 (11.9%) for T-PIT and 477 cases for SF-1 (51.8%). Of the remaining 9 cases (1%), one was confirmed as a double PA/PitNET, exhibiting positive immunostaining for PIT-1 and T-PIT in two distinct tumor components. Only 6 cases (0,7%) were completely negative for TF immunostaining, including negativity for ERα and GATA3, indicating that these were true null cell tumors. Two cases were classified as plurihormonal PA/PitNET. The addition of GATA3, ERα and CAM5.2 according to WHO 2022 also allows for the subdivision of plurihormonal PIT-1 positive PAs into mature and immature PIT-1-lineage tumors. Figure 1 illustrates the distribution of the 921 cases based on the WHO classification of 2017 (Fig. 1A) and 2022 (Fig. 1B), with an additional differentiation into sparsely and densely granulated subgroups where applicable.

### Transition from WHO classification 2004 to WHO classification 2017

**Fig. 2:**
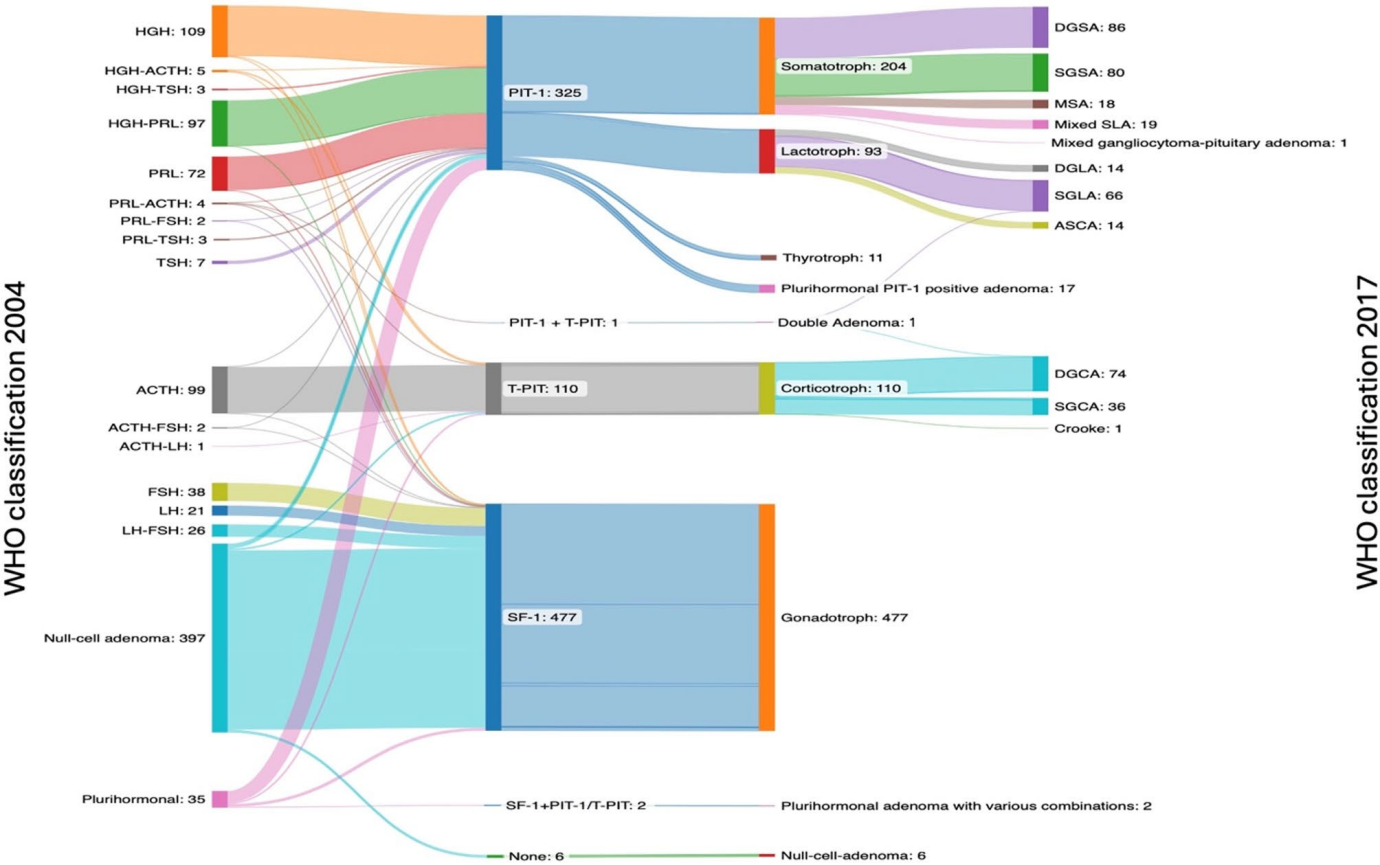
Transition of histopathological classification from WHO classification 2004 (left) to 2017 (right)

The most significant innovation of WHO classification 2017 was the introduction of TF. Consequently, the classifications of 2004 and 2017 were compared, and shifts in histopathological (sub)types were analyzed (Fig. 2).

### Transition from WHO classification 2004 to WHO classification 2017 and clinico-pathological correlation: PAs/PitNETs formerly classified as null cell adenomas

**Fig. 3:**
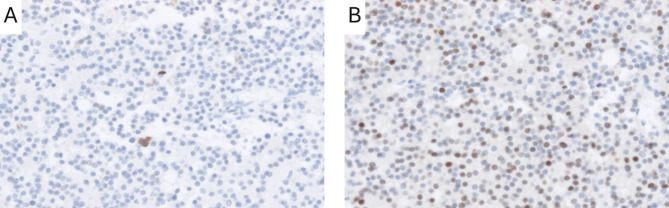
Example for transition between 2004 and 2017. Tumor classified as a null cell adenoma with irrelevant ACTH expression according to WHO classification 2004 (**A**), with the addition of strong T-PIT in the nuclei, reassessed as silent corticotroph adenoma according to WHO classification 2017 (**B**)

**Fig. 4:**
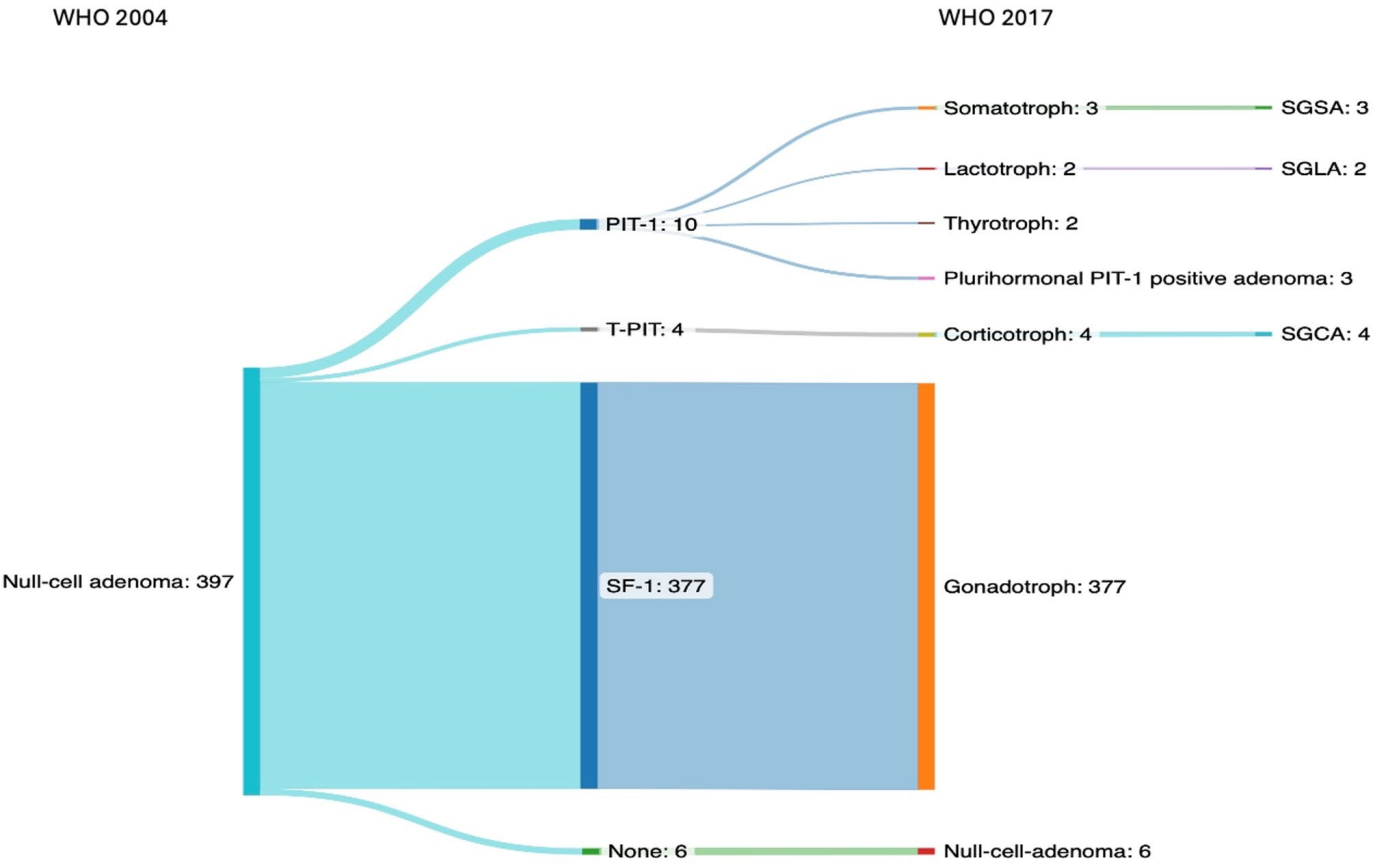
Overview of reclassification of null cell adenomas

According to the 2004 WHO classification, null cell adenomas represented the largest subgroup with a total of 397 cases (43.1%). With the application of the 2017 WHO classification, 377 of the 397 cases (95%) null cell adenomas were reclassified as gonadotroph PAs/PitNETs due to positive SF-1 immunostaining in tumor nuclei, 10 cases (2.5%) were reassigned to the PIT-1 lineage (3 SGSA, 2 SGLA, 2 thyrotroph, 3 plurihormonal PIT-1 positive adenomas), and 4 cases (1%) were reclassified as T-PIT lineage cases (4 SGCA, Fig. 3). Only 6 cases (1.5%) remained null cell adenomas (Fig. 4).

Among the SF-1 lineage PAs/PitNETs, no significant clinical or endocrinological signs of hormone excess were observed in 369 of 377 cases (97.9%). Of the remaining 8 patients with SF-1 lineage tumors, 4 had secondary amenorrhea, one had gynecomastia, one had clinical signs of acromegaly and one suffered from hyperhidrosis as possible signs of hormonal activity. However, these symptoms were not associated with a corresponding hormone excess. The eighth patient was the only one with confirmed hormone overproduction. She was a 61 years old patient with a 27 mm intra- and suprasellar macroadenoma, with typical stigmata and clinical symptoms of acromegaly that was confirmed endocrinologically (IGF-1: 640.0 ng/ml; hGH: 3.81 µg/L) and was in remission postoperatively (IGF-1: 135.0 ng/ml; hGH: 0.37 µg/L). Re-analysis of this case found no evidence of PIT-1 co-expression.

Of the 14 cases that were reassessed to PIT-1 lineage (*n* = 10) or T-PIT lineage (*n* = 4), 6 patients had concomitant slight prolactinemia (2-3-fold). A fivefold increase in PRL was observed in only one of the reassessed SGLA cases. Following surgery, the prolactin level was always within the normal range. In these 14 cases, retrospective IHC revealed focal hormone expression in less than 1% of tumor cells. At the time of original diagnosis, the absence of defined thresholds for hormone positivity likely contributed to their classification as null cell adenomas. However, TF analysis demonstrated clear lineage-specific expression (e.g., PIT-1, T-PIT), supporting their reclassification.

The 6 confirmed true null cell adenomas demonstrated neither clinical nor biochemical evidence of any hormonal activity.

### Transition from WHO classification 2004 to WHO classification 2017 and clinicopathological correlation: formerly classified as plurihormonal PAs/PitNETs

**Fig. 5:**
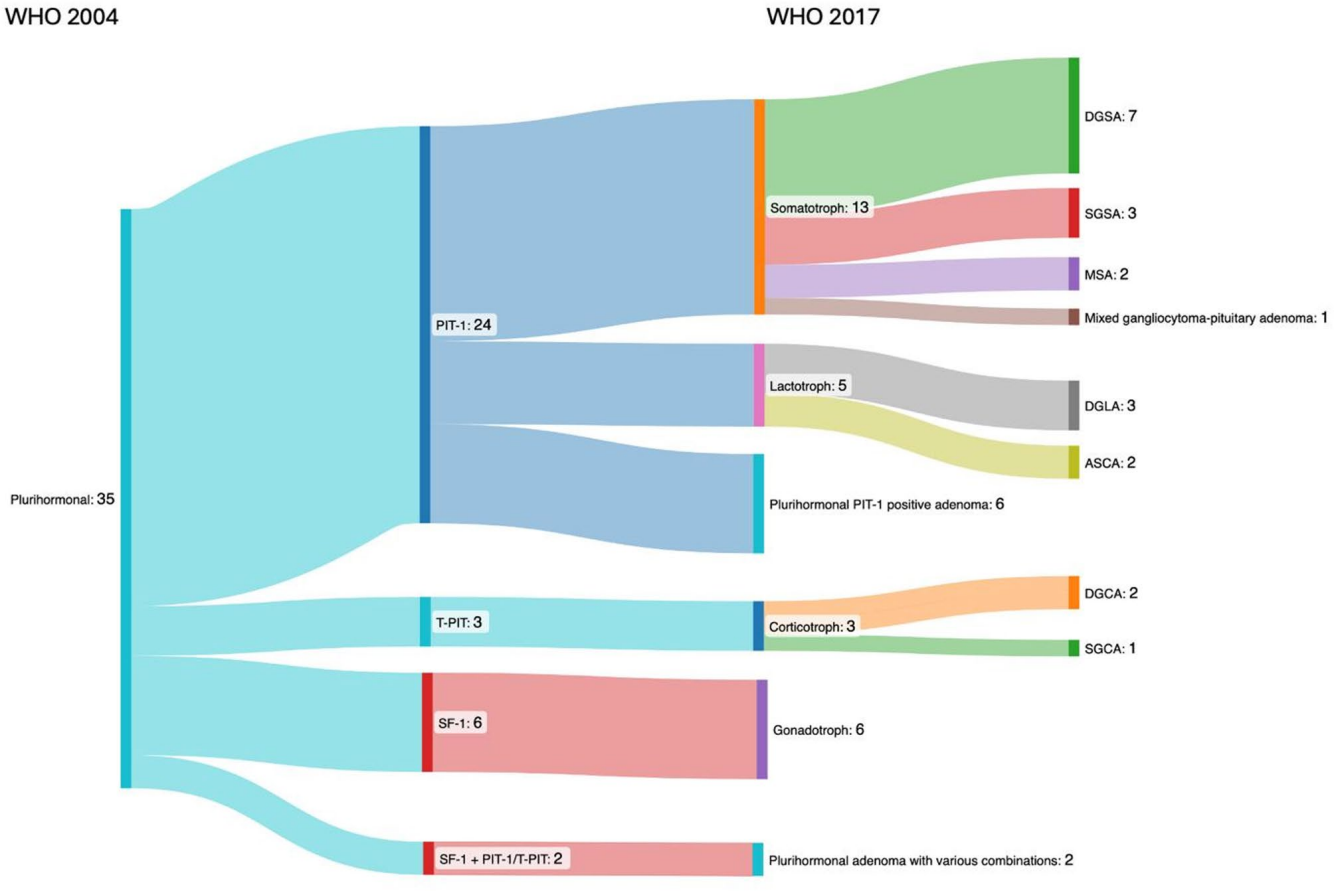
Overview reclassification of plurihormonal adenomas

Of the 35 plurihormonal adenomas (3.8%) according to the WHO classification of 2004, 33 cases (94.3%) could be assigned to a specific TF lineage. Twenty-four cases (68.6%) were positive for PIT-1: 13 belonged to the somatotroph subgroup (7 DGSA, 3 SGSA, 2 MSA, 1 mixed gangliocytoma-somatotroph PitNET), 5 to the lactotroph subgroup (3 DGLA, 2 ASCA), and 6 remained classified as plurihormonal PIT-1 positive adenomas. Three cases (8.6%) were assigned to the T-PIT lineage, while 6 cases (17.1%) were assigned to the SF-1 lineage. The remaining 2 cases (5.7%) were classified as plurihormonal PA/PitNET with various combinations (Fig. 5).

In the somatotroph group according to WHO 2017 (Fig. 5), only one patient with a SGSA was observed to be clinically and laboratory nonfunctioning, while the other 12 exhibited endocrinologically confirmed acromegaly. In the group of lactotroph PA/PitNET, all patients with DGLAs had hyperprolactinemia and corresponding clinical symptoms. Both ASCAs were clinically non-functioning, although one of them was suspected to have a slight TSH release. Four of 6 patients with plurihormonal PIT-1 positive tumors had clinical and biochemical evidence of acromegaly, one patient suffered Cushing’s disease (CD), and one was non-functioning. Of the three corticotroph adenomas, one had caused CD, while the other two were classified as silent corticotroph adenomas. However, one of them then developed CD over time. The 6 SF-1 positive cases showed neither clinical nor laboratory signs of hormonal activity, consistent with non-functioning gonadotroph PA/PitNET. The two plurihormonal PA/PitNET with various combinations corresponded clinically to a confirmed acromegaly and a confirmed CD, respectively.

### Transition from WHO classification 2004 to WHO classification 2017 and clinicopathological correlation: functional PAs/PitNETs

**Fig. 6:**
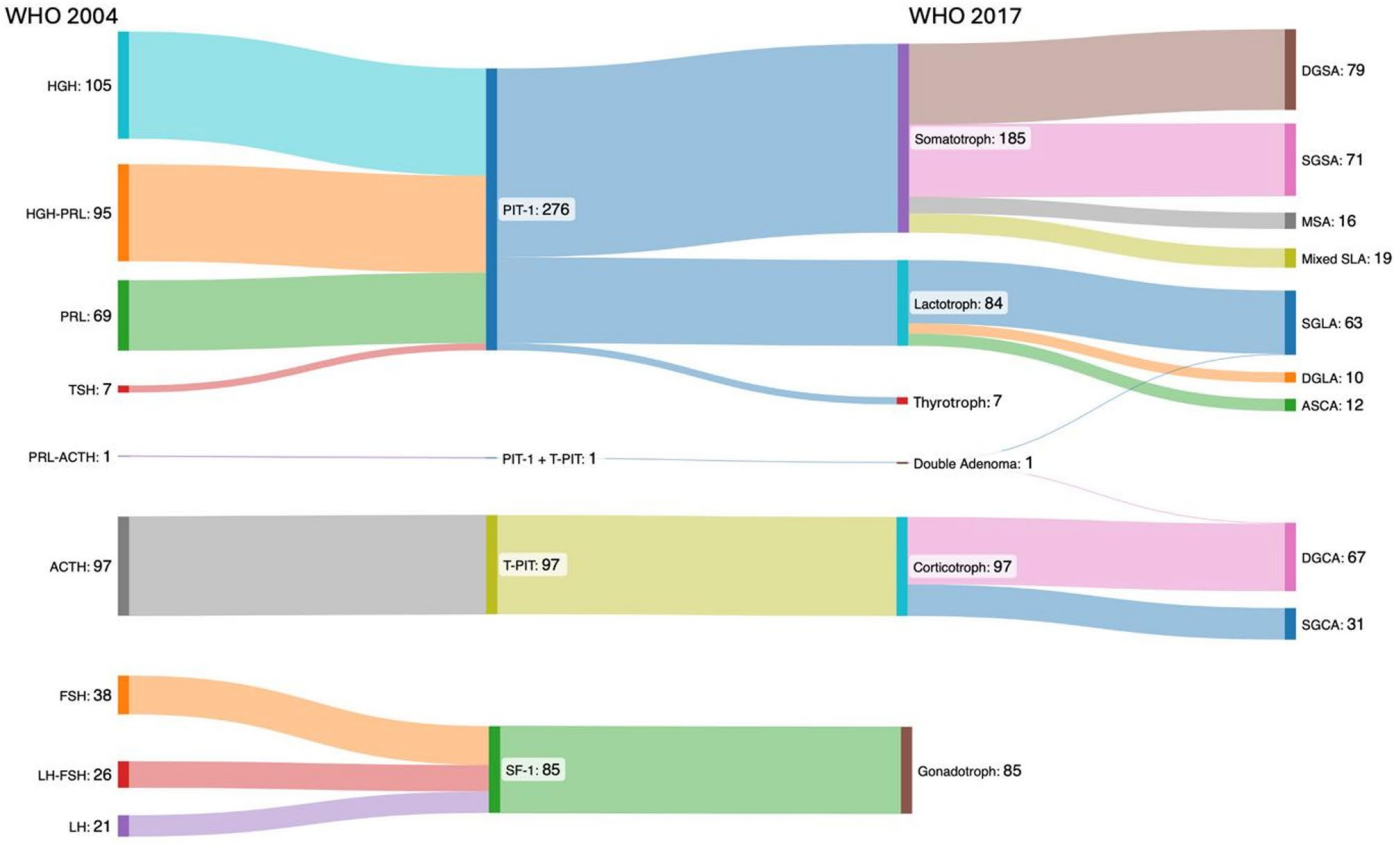
Overview adenomas with expression of one/two hormones and confirmed classification in same cell lineage

According to the WHO classification of 2004, a total of 489 PAs (53.1%) was categorised as functional adenomas expressing one or two hormones. In 459 of these cases (93.9%), the corresponding cell transcription lineage was confirmed, with one instance of a double adenoma expressing PIT-1 (SGLA subtype) and T-PIT (DGCA subtype), respectively (Fig. 6).

According to the WHO classification 2017, the hormone activity identified by TF and immunohistochemistry was confirmed clinically and endocrinologically in 444 of 459 cases (96.7%). The remaining 15 cases (3.3%) had neither clinical nor laboratory evidence of hormone secretion (6 DGSA, 2 SGSA, 1 DGLA, 2 SGLA, 2 thyrotroph, 1DGCA, 1 SGCA). In the case of the double adenoma, a CD was confirmed and clearly in the foreground, and laboratory tests also confirmed a significant elevation in PRL levels (> 100-fold). All SF-1 positive adenomas were confirmed to be clinically and biochemically non-functioning.

**Fig. 7:**
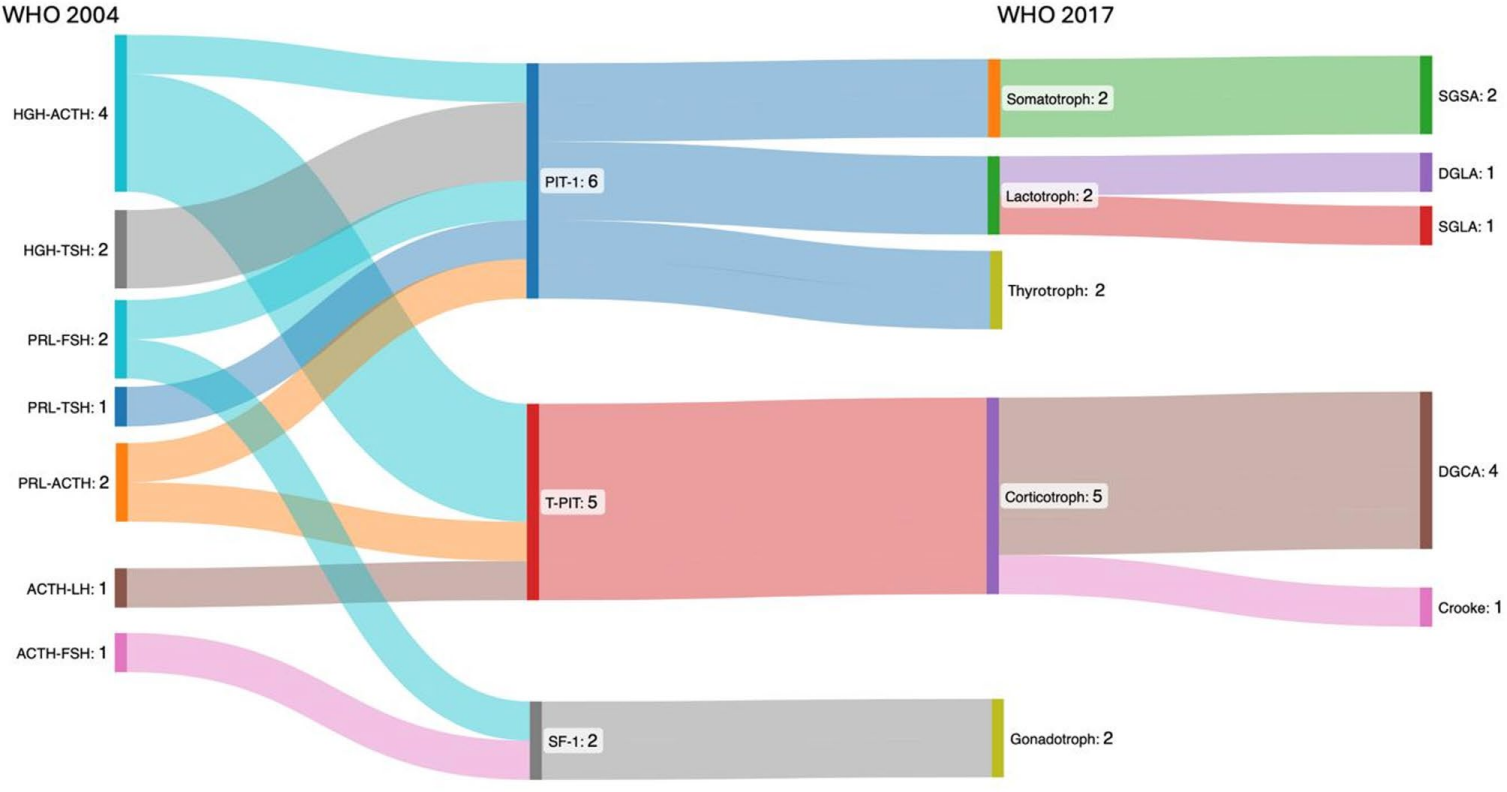
Overview of adenomas with expression of two hormones and reclassification to one specific cell lineage

Thirteen cases (2.7%) with previous described immunohistochemical expression of two hormones derived from two distinct cell lineages, could be assigned to a single transcription cell lineage (Fig. 7).

A re-evaluation of the clinical data confirmed the respective reassessed cell lineage. The two patients with SGSAs had acromegaly, two patients with lactotroph adenomas had clinical and biochemical evidence of prolactin secretion, and of the 5 patients with corticotroph PitNETs/PAs, 4 had confirmed CD. The remaining T-PIT positive case was reassessed as a silent corticotroph adenoma. Both PAs/PitNETs with ACTH and FSH expression were clinically non-functioning.

Three cases (0.6%) with expression of two hormones derived from the PIT-1 lineage, could be assigned to one distinct subtype. Of the two PAs/PitNETs expressing GH and TSH, one was reclassified as SGSA and the other as thyrotroph. Additionally, one PA/PitNET expressing PRL and TSH was reclassified as thyrotroph (Fig. 7). The reclassification of these cases was also consistent with the clinical data: one patient diagnosed with acromegaly and two patients with hyperthyroidism.

**Fig. 8:**
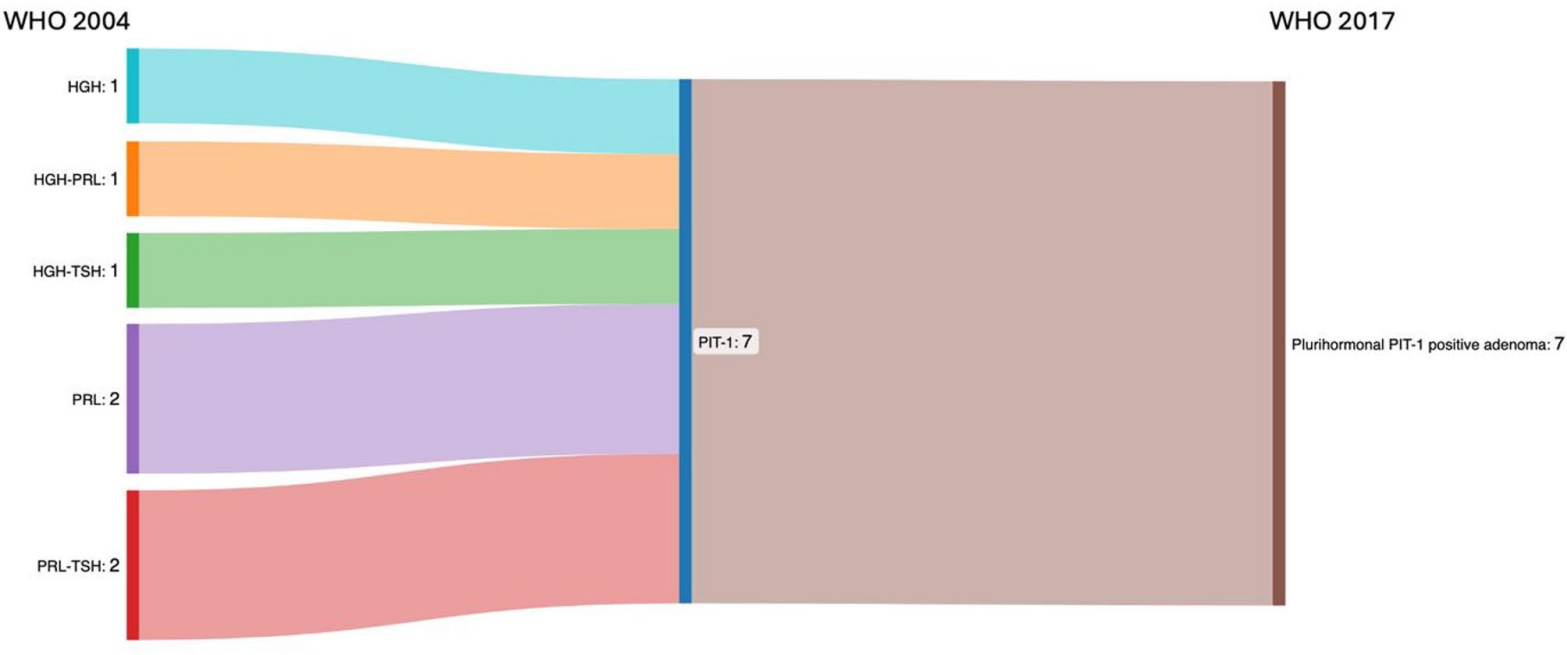
Overview adenomas with one or two hormone expression and reclassification as plurihormonal PIT-1 positive adenomas

Seven cases (1.4%) with expression of 1 or 2 hormones of the PIT-1 lineage (HGH, PRL, TSH) were reclassified as plurihormonal PIT-1 positive adenomas (Fig. 8). It is noteworthy, that three cases previously classified as only GH- or PRL-producing adenomas were reclassified as PIT-1-positive plurihormonal adenomas. In all three tumors, the majority of cells showed strong expression of either GH or PRL. However, additional focal TSH expression was detected in each case, and in one tumor, sparse ACTH-positive cells were also observed. Despite the limited extent of these secondary hormone expressions, the co-expression of multiple hormones—together with diffuse nuclear PIT-1 positivity—fulfilled the criteria for classification as PIT-1-positive plurihormonal adenomas.

A review of these seven patient records revealed that five of these cases were nonfunctioning, one had acromegaly and one hyperthyroidism.

**Fig. 9:**
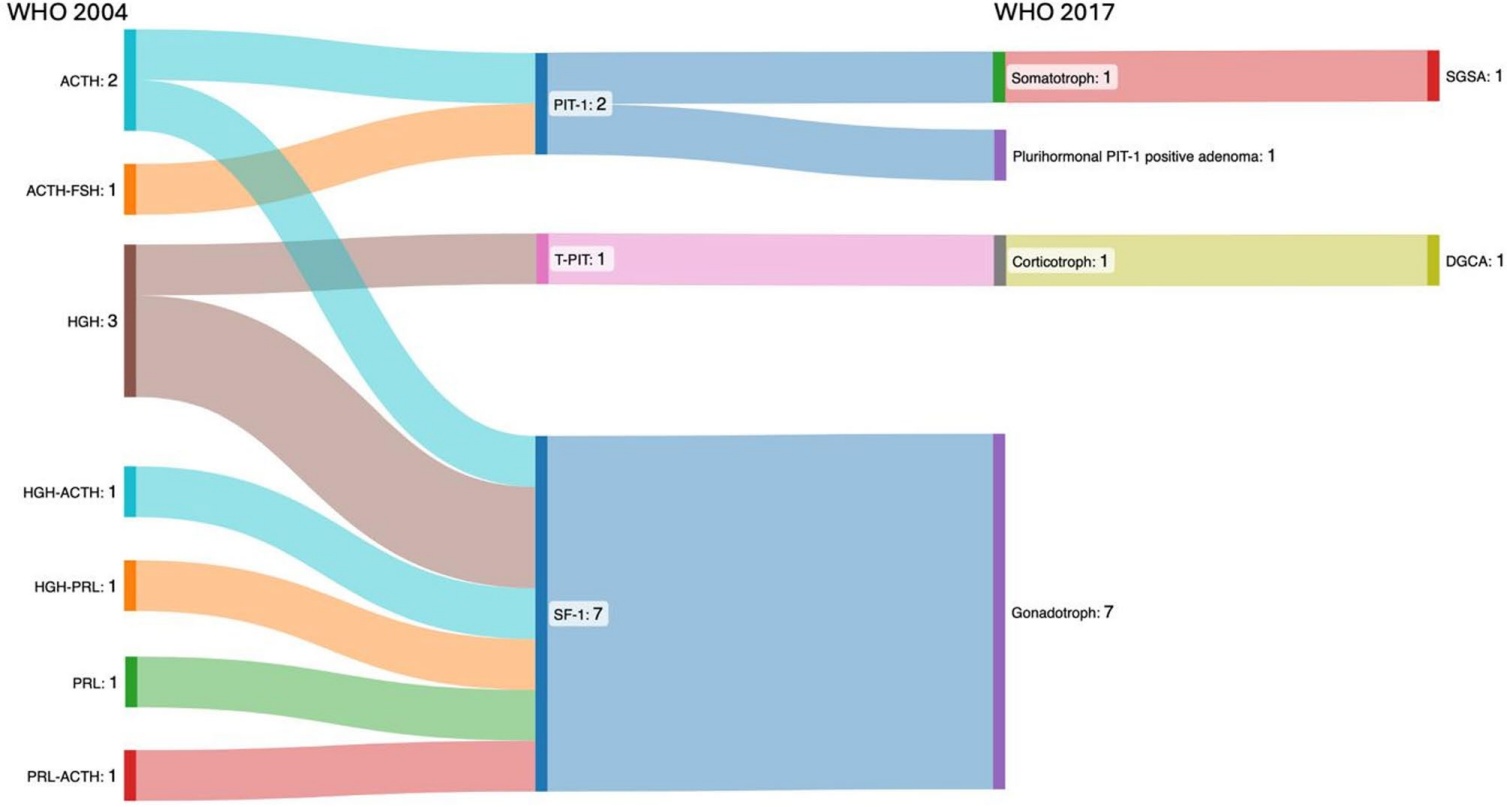
Overview reclassification to a different cell lineage

The remaining 10 cases (2%) were reclassified into a different TF lineage (Fig. 9).

Seven cases (2 HGH, 1 HGH + ACTH, 1 HGH + PRL, 1 PRL, 1 PRL + ACTH and 1 ACTH) were SF-1 positive and thus reassessed as gonadotroph. All were clinically and laboratory nonfunctioning, exhibiting a low number of cells with hormone expression, highly suggestive of entrapped non-neoplastic pituitary cells. One previously described HGH-expressing adenoma was reclassified as DGCA (T-PIT lineage) and was confirmed to have a clinically confirmed CD. One ACTH-expressing adenoma was classified as SGSA (PIT-1 lineage). Despite the absence of hormonal abnormalities, this case presented with clinical symptoms of hyperhidrosis and hypertonia. One ACTH- and FSH-expressing adenoma was reassessed as plurihormonal PIT-1 positive adenoma (Fig. 9). This case was clinically and laboratory nonfunctioning.

### Transition from WHO classification 2017 to WHO classification 2022

While the implementation of transcription factor-based classification in the WHO classification 2017 guidelines represented the most significant diagnostic shift, the subsequent WHO classification 2022 introduced several important refinements. These primarily involved a more nuanced subclassification of PIT-1-lineage tumors and the incorporation of additional immunohistochemical markers– ERα, GATA3, and CAM5.2– to improve characterization of diagnostically ambiguous cases. According to the WHO classification 2022, some PIT-1 positive tumors are no longer categorized solely as somatotroph, lactotroph or thyrotroph PA/PitNETs, but are now stratified into distinct types, including mammosomatotroph, mixed somatotroph–lactotroph, and acidophil stem cell PA/PitNETs (Fig. 1).

In our cohort, the application of the WHO 2022 criteria did not result in major shifts in overall tumor distribution when compared to the 2017 classification. All six tumors previously identified as null cell adenomas remained classified as true null cell PitNETs, even after extended immunohistochemical evaluation with ERα, GATA3, and CAM5.2. The most notable change was observed in the subdivision of PIT-1 positive plurihormonal tumors into mature (*n* = 11) and immature (*n* = 6) types. The mature PIT-1-positive plurihormonal PitNETs exhibited strong expression of one or more PIT-1-regulated hormones (GH, PRL, and/or TSH), and consistently showed positivity for ERα and GATA3, with a perinuclear staining pattern for CAM5.2. In contrast, the immature PIT-1-positive tumors demonstrated much weaker hormone expression, with only 2 of 6 cases showing GATA3 positivity and 1 case showing ERα positivity. CAM5.2 staining was diffusely positive across the cells in this group.

The two cases, previously classified as plurihormonal adenomas with various combinations, were now defined as plurihormonal PitNETs. One case showed co-expression of SF-1 and PIT-1, along with hormone positivity for LH, PRL, and GH. Clinically, this case corresponded to acromegaly. The second case showed SF-1 and T-PIT co-expression and was positive for ACTH and FSH, with clinical features consistent with Cushing’s disease.

### Clinicopathological correlation after TF-based reclassification

Overall, a correct correlation was observed between the clinical and laboratory data and the WHO classification of 2004 in 75.4% (*n* = 694) of cases. Following the addition of TF, the correlation increased to 96.2% (*n* = 886). The introduction of TF resulted in a more accurate classification of the underlying PA/PitNET type compared to the previous classification. The improved correlation of histopathology and clinical data was highly significant (*p* <.0001). A total of 432 nonfunctioning PAs/PitNETs were identified through clinical and laboratory data. These were primarily reflected in the SF-1 distinct cell lineage (*n* = 384; 88.9%). The remaining 48 cases were distributed among the other cell lineages: 33 (7.6%) were PIT-1 positive, 9 (2.1%) were T-PIT positive, and 6 (1.4%) were identified as null-cell tumors.

### Recurrence and progression free survival

**Fig. 10:**
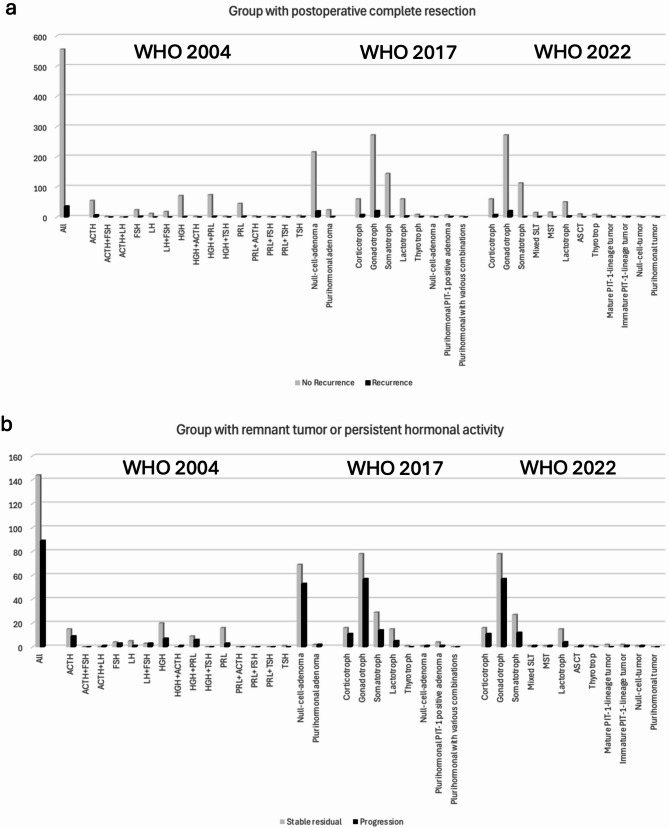
Distribution of recurrence (**a**) and progression (**b**) according to the WHO classifications

For survival analysis, 82 patients without FU and 14 patients with only postoperative CT imaging were excluded. The remaining cohort (*n* = 825) was divided into 2 groups: the first group with postoperative complete resection and, if preoperative hormonal active, biochemical remission (*n* = 592), and the second with postoperative residual tumor or persistent hormone overproduction (non-remission) (*n* = 233). In the first groups, only 36 patients (6.1%) developed a recurrence, whereas in the second group 89 patients (38.2%) had a progression. Figure 10 shows the distribution of recurrence and progression among the WHO classification cohorts.

Using a Kaplan-Meier analysis, we visualized the recurrence-free (Table 3) and progression-free (Table 4) survival probability of the respective PA/PitNET types reflecting the changes based on the different WHO classifications.

**Table 3:** Recurrence-free survival according to WHO classifications 2004, 2017 and 2022

	No Recurrence (*n*=)	Recurrence (*n*=)	Recurrence Development Mean (months)	Recurrence-free Survival Probability 5 years after surgery	Recurrence Probability 5 years after surgery	Recurrence-free Survival Probability 10 years after surgery	Recurrence Probability 10 years after surgery
All	*556*	*36*	*185.8*	*98.3%*	*1.7%*	*89.9%*	*10.1%*
**WHO 2004**							
ACTH	*55*	*7*	*99.3*	*93.6%*	*6.4%*	*61.3%*	*38.7%*
HGH	*71*	*1*	*40*	*98.6%*	*1.4%*	*98.6%*	*1.4%*
HGH + PRL	*74*	*2*	*115*	*100%*	*0%*	*93.0%*	*7.0%*
PRL	*45*	*2*	*130.8*	*97.9%*	*2.1%*	*92.1%*	*7.9%*
TSH	*5*	*1*	*35*	*83.3%%*	*1.7%*	*83.3%*	*1.7%*
Null-cell-adenoma	*215*	*20*	*183.6*	*99.1%*	*0.9%*	*89.7%*	*10.3%*
Plurihormonal adenoma	*24*	*1*	*33*	*96%*	*4%*	*96%*	*4%*
Others (ACTH + FSH; ACTH + LH; FSH; LH; LH + FSH; HGH + ACTH; HGH + TSH; PRL + ACTH; PRL + FSH)	*67*	*2*	*107*	*100%*	*0%*	*95.5%*	*4.5%*
**WHO 2017**							
Corticotroph	*60*	*8*	*98.9*	*92.7%*	*7.3%*	*61.7%*	*38.3%*
Gonadotroph	*272*	*21*	*183.5*	*99.3%*	*0.7%*	*89.5%*	*10.5%*
Somatotroph	*144*	*2*	*115.5*	*99.3%*	*0.7%*	*94.6%*	*5.4%*
Lactotroph	*60*	*3*	*129.9*	*98.4%*	*1.6%*	*96.2%*	*3.8%*
Thyrotroph	*9*	*1*	*35*	*90%*	*10%*	*90%*	*10%*
Null-cell-adenoma	*2*	*0*	*-*	*100%*	*0%*		
Plurihormonal PIT-1 positive adenoma	*7*	*1*	*80*	*100%*	*0%*	*75%*	*25%*
Plurihormonal with various combinations	*2*	*0*	*-*	*100%*	*0%*		
**WHO 2022**							
Corticotroph	*60*	*8*	*98.9*	*92.7%*	*7.3%*	*61.7%*	*38.3%*
Gonadotroph	*272*	*21*	*183.5*	*99.3%*	*0.7%*	*89.5%*	*10.5%*
Somatotroph	*113*	*1*	*40*	*99.1%*	*0.9%*	*99.1%*	*0.9%*
Mixed SLT	*15*	*1*	*116*	*100%*	*0%*	*83.3%*	*16.7%*
MST	*16*	*0*	*-*	*100%*	*0%*	*100%*	*0%*
Lactotroph	*50*	*3*	*129.3*	*98.1%*	*1.9%*	*95.5%*	*4.5%*
ASCT	*10*	*0*	*-*	*100%*	*0%*		
Thyrotroph	*9*	*1*	*35*	*90%*	*10%*	*90%*	*10%*
Mature PIT-1-lineage tumor	*5*	*0*	*-*	*100%*	*0%*		
Immature PIT-1-lineage tumor	*2*	*1*	*80*	*100%*	*0%*	*50%*	*50%*
Null-cell-tumor	*2*	*0*	*-*	*100%*	*0%*		
Plurihormonal tumor	*2*	*0*	*-*	*100%*	*0%*		

**Table 4:** Progression-free survival according to WHO classification 2004, 2017 and 2022

	Residual stable (*n*=)	Progression (*n*=)	Progression Development Mean (months)	Progression-free Survival Probability 5 years after surgery	Progression Probability 5 years after surgery	Progression-free Survival Probability 10 years after surgery	Progression Probability 10 years after surgery
All	*144*	*89*	*109.8*	*72.0%*	*28.0%*	*51.2%*	*48.8%*
**WHO 2004**							
ACTH	*15*	*9*	*67.9*	*66.7%*	*33.3%*	*55.6%*	*44.4%*
HGH	*20*	*7*	*82.5*	*81.5%*	*18.5%*	*59.8%*	*40.2%*
HGH + PRL	*9*	*6*	*79.4*	*73.3%*	*26.7%*	*48.9%*	*51.1%*
PRL	*16*	*3*	*95.5*	*89.5%*	*10.5%*	*67.1%*	*32.9%*
TSH	*1*	*0*	*-*	*100%*	*0%*		
Null-cell-adenoma	*69*	*53*	*103.2*	*68.9%*	*31.1%*	*40.6%*	*59.4%*
Plurihormonal adenoma	*2*	*2*	*48.8*	*75%*	*25%*	*50%*	*50%*
Others (ACTH + LH; FSH; LH; LH + FSH; HGH + ACTH),	*12*	*9*	*67.2*	*66.7%*	*33.3%*	*48.6%*	*51.4%*
**WHO 2017**							
Corticotroph	*16*	*11*	*66.9*	*62.9%*	*37.1%*	*52.5%*	*47.5%*
Gonadotroph	*78*	*57*	*104.8*	*70.4%*	*29.6%*	*41.3%*	*58.7%*
Somatotroph	*29*	*14*	*81.2*	*76.7%*	*23.3%*	*53%*	*47%*
Lactotroph	*15*	*5*	*88*	*85%*	*15%*	*60%*	*40%*
Thyrotroph	*1*	*0*	*-*	*100%*	*0%*		
Null-cell-adenoma	*1*	*1*	*16*	*50%*	*50%*	*50%*	*50%*
Plurihormonal PIT-1 positive adenoma	*4*	*1*	*36*	*80%*	*20%*	*80%*	*20%*
**WHO 2022**							
Corticotroph	*16*	*11*	*66.9*	*62.9%%*	*37.1%*	*52.5%*	*47.5%*
Gonadotroph	*78*	*57*	*104.8*	*70.4%*	*29.6%*	*41.3%*	*58.7%*
Somatotroph	*27*	*12*	*82.3*	*79.5%*	*20.5%*	*54.6%*	*45.4%*
Mixed SLT	*1*	*1*	*57*	*50%*	*50%*		
MST	*1*	*1*	*28*	*50%*	*50%*	*50%*	*50%*
Lactotroph	*15*	*4*	*91.8*	*84.2%*	*15.8%*	*63.2%*	*36.8%*
ASCT	*0*	*1*	*15*	*0%*	*100%*	*0%*	*100%*
Thyrotroph	*1*	*0*	*-*				
Mature PIT-1-lineage tumor	*2*	*0*	*-*	*100%*	*0%*		
Immature PIT-1-lineage tumor	*2*	*1*	*36*	*66.7%*	*33.3%*		
Null-cell-tumor	*1*	*1*	*16*	*50%*	*50%*	*50%*	*50%*

Following complete resection, the risk of recurrence is minimal, with a recurrence-free survival rate of 98.3% 5 years post-surgery, and of 89.9% 10 years after surgery. The corticotroph group showed the highest risk of recurrence, at 38.3% within the ten-year interval. In cases where residual PA/PitNET or residual activity is present, only 51.2% of cases demonstrate stable image findings 10 years after surgery. The gonadotroph and corticotroph group carried the greatest risk of progression, with respectively a probability of progression of 29.6% and 37.1% 5 years after surgery, and 58.7% and 47.5% 10 years after surgery. A comparison between the WHO classification of 2017 and 2022 revealed no significant survival differences between the newly independent types MSLT, MST, and ASCT (previously a subgroup of the somatotroph and lactotroph group). However, these groups were relatively small, limiting statistical power. Despite this limitation, the analysis revealed that among the 2017 plurihormonal PIT-1 positive adenomas, according to the WHO classification 2022, only the immature PIT-1 lineage tumors showed recurrence and progression, in contrast to the mature PIT-1 lineage tumors.

## Discussion

The classification of PAs, now termed PitNETs has undergone a significant transformation since the inception of the WHO classification of tumors of endocrine organs. Initially, conventional light microscopy with HE and PAS-Orange-G staining was employed, which was subsequently supplemented by immunostaining for pituitary hormone expression and ultrastructural features of the tumor cells. The introduction of immunohistochemistry led to a significant advancement in the classification of hormonal activity in functioning adenomas [1, 3, 20]. The use of specific pituitary TF has enabled a more precise classification of PA/PitNET and has improved correlation to clinical presentation [14, 18]. Our analysis confirmed these findings in the largest group published to date. Of the 921 eligible patients, the WHO classification 2004 retrospectively demonstrated a correct correlation of 75.4% with clinical and endocrinological findings, while the introduction of TF increased this rate to 96.2%. Of course, it remains of utmost importance to consider the clinical and endocrinological findings when determining the diagnosis and the subsequent therapy for the patient.

Our data also confirm that almost all PitNETs can be assigned to distinct lineages of pituitary cells with the use of TF. In our large cohort, only 6 cases (0.7%) remain hormone-negative and transcription factor negative and are still classified as null cell tumors. It is noteworthy that some null cell adenomas according to the 2004 WHO classification could be reclassified in a PIT-1 (*n* = 10) or T-PIT (*n* = 4) subtype. In these cases, retrospective IHC revealed minimal hormone expression (< 1% of tumor cells), which at the time was not considered sufficient for functional classification—particularly in the absence of defined cut-off values. These tumors could now be reassigned to specific hormonal cell lineages and classified as “silent” subtypes (e.g., SGSA, SGLA, SCGA). These findings highlight the diagnostic limitations of earlier classification systems and underscore the importance of combining hormone and TF profiling for accurate tumor classification. The diagnostic work-up of PAs/PitNETs should therefore include an analysis of all three TFs in previously non-conclusive cases. On the other hand, the analysis of hormone staining cannot be omitted and remains mandatory to correctly identify plurihormonal PAs/PitNETs.

The application of the WHO classifications 2017 and 2022 reveals a notable shift in the distribution of PAs/PitNETs, as illustrated in Fig. 2. Importantly, the null cell adenomas according to the old 2004 WHO classification are now almost entirely assigned to the gonadotroph group (SF-1 positive or GATA3 and ERα). In our cohort, the classification of clinically non-functional PAs/PitNETs is as follows: the majority of cases (88.9%) were classified as belonging to the SF-1 cell lineage, while 7.6% were assigned to the PIT-1 lineage, 2.1% to the T-PIT lineage, and only 1.4% were identified as true null cell tumors. In contrast, other authors present a different distribution of the “silent” non-functioning PAs/PitNETs. In the literature, the gonadotroph PitNETs/PAs consistently represent the largest group, and the silent corticotroph PAs/PitNETs is the second most frequent group [21–23].

In our cohort, we were able to confirm the significantly less prevalent occurrence of null cell PAs/PitNETs [16, 21, 22], with only 6 (0.7%) null cell tumors remaining. This change can be attributed to two factors: the introduction of TF and the absence of a clear cut-off for TF immunopositivity. Nishioka et al. described that > 80% of nuclei within a tumor must be positive for TP [22, 24]. Indeed, other authors suggest that a small number of positive nuclei is sufficient for a correct histological type diagnosis [15, 25]. In cases with weak SF-1 positivity, correct classification can be further supported by GATA3 and ERα stains. It remains unclear whether the null cell PAs/PitNETs will retain their designation as such, or whether future studies may identify other factors that reclassify them in a distinct cell lineage, potentially leading to their complete disappearance from the subgroup classification [24]. In recent years research in DNA-sequencing and methylation profiling for PAs/PitNETs increased [24, 26, 27]. Eventually this could be the next method for finally classifying the remaining unclear null cell tumors either as a distinct group or reassigning these non-functioning tumors to an existing cohort. This need for further refinement is also especially evident in rare cases where the histological and clinical diagnosis diverge. For example, in one of our patients with confirmed acromegaly, the tissue was SF-1 positive but completely negative for PIT-1 and T-PIT. Such findings underscore the potential value of DNA-sequencing and methylation profiling in clarifying lineage assignment in borderline or discordant cases.

Due to the numerous different subtypes of PAs/PitNETs, an exact histopathological examination is required to ensure a correct clinicopathological correlation. A precise histopathological classification is paramount to plan follow-up and further treatment, and to predict the patients’ prognosis. According to the 2004 WHO classification, the criteria for high-risk adenomas (“atypical adenomas ”) were a Ki-67 proliferation index of 3% or more, an extensive nuclear staining for p53 protein (often defined as expression of 10% positive nuclei or more), an increased rate of mitosis and their invasive growth [2, 3, 28]. However, the definition of atypical adenomas has been abandoned because its low predictive value. It has been shown that certain tumor types or subtypes are associated with an adverse biological behavior and they are now defined as high-risk PAs/PitNETs according to the 2017 and 2022 WHO classifications, such as male lactotroph, silent corticotroph and Crooke cell, sparsely granulated somatotroph, and silent plurihormonal PIT-1 positive tumors [12, 13, 15, 16, 18]. Our study confirms that corticotroph PAs/PitNETs/ are particularly prone to recurrence and progression, as described in the literature [12, 15, 16, 18]. However, the number of cases included in some groups is very small, which makes it challenging to draw meaningful conclusion. For example, our evaluation does not provide evidence of a significant distinction between the refinement of MST, MSLT and ASCT considered as a distinct tumor type, in comparison to the previously merged subgroup into the somatotroph and lactotroph type. Following the assumption of the latest WHO classification 2022, we see in our small subgroups that recurrence and progression occurs only in the group of immature tumors of the PIT-1 lineage and not of the mature ones [15, 18].

A precise neuropathological classification of PitNETs is of great importance for determining the risk of recurrence or progression, as well as for guiding further therapeutic interventions. For example, in patients with acromegaly, a better response to somatostatin analogues has been observed in the dense granulated somatotroph PitNETs/PAs in comparison to the sparsely granulated [28]. This emphasizes the importance of keratin immunostains in determining tumor subtype in somatotrophs [29]. In individual cases, the change in the histopathological diagnosis resulting from the reclassification must be taken into account in the further patient management and FU planning. Further subgroup analyses, also with regard to the response to drug and radiotherapy treatment, are necessary in order to enable the best monitoring of the patients with PitNETs/PAs according to the histopathological types and subtypes.

## Limitations

The most prominent limitation is a selection bias based on the composition of this single center surgical cohort. While this cannot be controlled for, it needs to be kept in mind when comparing the results with other cohorts. Conversely, the principal strength of the single-centre study is that the tumor tissue was obtained and analysed in an uniform manner for all samples.

Some cases had to be excluded from the reclassification due to insufficient tumor tissue or inadequate staining. Nevertheless, a sufficient cohort of 921 cases was compiled.

Another limitation of the study is the use of TMAs. PitNETs can be heterogeneous, especially if they express more than one hormone. We used representative areas of the tumor, but the TMAs are still less representative than the whole tumor.

With regard to the clinical data, the main limitation is the retrospective nature of the study. While there are no gaps in the preoperative data and therefore an accurate assessment of the clinical and laboratory status is available, there was a heterogeneous collection of data, particularly in the investigation of the long-term follow-up. A total of 96 cases also had to be excluded because of missing follow-up data. As the overall cohort spans 14 years, the entire cohort could be included into the 5-year FU post-surgery. For each additional year, a part of the total cohort was censored. Additionally, the number of cases included in some subgroups is very small, which makes it challenging to draw meaningful conclusions. The FU analysis only considered whether and when a recurrence or progression occurred. A further analysis regarding the further therapy and its response has not been evaluated.

## Conclusion

The introduction of transcription factors and markers such as GATA3 and ERα significantly improved the correlation between histopathological classification and clinical presentation (from 75.3 to 96.2%). Most previously classified null cell adenomas were redefined as gonadotroph PitNETs, while true null cell and plurihormonal PitNETs were found to be far less common than previously assumed. Although our results confirm existing knowledge, the strength of this study lies in its large, uniformly analyzed cohort and the integration of long-term clinical outcomes. Our findings underline the clinical relevance of accurate classification for prognosis and patient management, particularly in identifying subtypes with higher recurrence risk, such as corticotroph PitNETs.

## Data Availability

All necessary data for this study are included in the manuscript. Additional data can be obtained from the correspnding author upon reasonable request.

## Abbreviations

ACTH: Adrenocorticotrophic hormone
ASCA: Acidophil stem cell adenoma
ASCT: Acidophil stem cell tumor
CD: Cushing disease
CT: Computer tomography
DGCA: Dense granulated corticotroph adenoma
DGCT: Dense granulated corticotroph tumor
DGLA: Dense granulated lactotroph adenoma
DGLT: Dense granulated lactotroph tumor
DGSA: Dense granulated somatotroph adenoma
DGST: Dense granulated somatotroph tumor
ER: Estrogen receptor
FSH: Follicle stimulating hormone
FU: Follow-up
GH: Growth hormone
HE: Haematoxylin and eosin
HGH: Human growth hormone
IGF-1: Insuline-like growth factor
LH: Luteinsing hormone
MSA: Mammosomatotroph adenoma
MSLA: Mixed somatotroph and lactotroph adenoma
MSLT: Mixed somatotroph and lactotroph tumor
MST: Mammosomatotroph tumor
PA: Pituitary adenoma
PIT-1: Pituitary-specific transcription factor-1
PitNET: Pituitary neuroendocrine tumors
PRL: Prolactin
SD: Standard deviation
SF-1: Steroidogenic factor-1
SGCA: Sparsely granulated corticotroph adenoma
SGCT: Sparsely granulated corticotroph tumor
SGLA: Sparsely granulated lactotroph adenoma
SGLT: Sparsely granulated lactotroph tumor
SGSA: Sparsely granulated somatotroph adenoma
SGST: Sparsely granulated somatotroph Tumor
T-PIT: T-box transcription factor
TF: Transcription factors
TMA: Tissue microassays
TSH: Thyroid stimulating hormone
WHO: World Health Organization
